# Radiological characterization of gilthead seabream (*Sparus aurata*) fat by X-ray micro-computed tomography

**DOI:** 10.1038/s41598-020-67435-2

**Published:** 2020-06-29

**Authors:** Diana Ceballos-Francisco, Nuria García-Carrillo, Alberto Cuesta, María Ángeles Esteban

**Affiliations:** 1 Immunobiology for Aquaculture Group, Department of Cell Biology and Histology, Faculty of Biology, University of Murcia, Campus Regional de Excelencia Internacional “Campus Mare Nostrum”, 30100 Murcia, Spain; 2 Preclinical Imaging Unit, Laboratory Animal Service, Core Facilities University of Murcia, 30120 Murcia, Spain

**Keywords:** Biological models, Zoology

## Abstract

In fish, the fat content contributes to promoting the nutritional and organoleptic characteristics of the flesh, which is crucial for consumer acceptance. Methods to predict the fat in fish are important in nutritional and physiological research, where body content is traditionally determined by dissection followed by chemical analysis. However, X-ray micro-computed tomography (micro-CT) provides three-dimensional information in a non-destructive way. This work aims to characterize radiologically the fat, in situ*,* in a widely cultivated marine species, gilthead seabream (*Sparus aurata*). To validate the method changes in fat content in a control group (fed) and another group (unfed for 60 days) were assessed. Fish images were acquired on an Albira SPECT/PET/CT preclinical-scanner. Image analysis and measurements were performed using the Carestream Molecular Imaging Albira CT system in conjunction with Pmod and Amide packages. By micro-CT analysis the density values were determined for the whole fish body (− 1,000 to + 2,500 HU, Hounsfield units), and density ranges for the fat in *S. aurata* were established from − 115 to + 50 HU. As expected, significant differences were found between fed and starved groups at 60 days. The present study confirms the usefulness of high-resolution morphological analysis for evaluating the presence and distribution of fat in this important fish species.

## Introduction

In recent decades, aquaculture has become an important social and economic activity and one of the largest sources of animal protein in the world. The success of modern aquaculture is based on the control of many parameters, among them: reproduction, a good knowledge of the biology of farmed fish, technological innovation and the development of specific feeds^1^. For example, a knowledge of fish biology is important to evaluate how the nutritional value of fish can be improved by controlling certain types of conditions. In this sense, knowledge of the fat content is essential for feeding, reproduction and genetic programs^2^. In addition, it is well known that fat contributes to the nutritional and organoleptic characteristics of fish flesh, which is crucial for consumer acceptance^2–6^. Advanced methods for predicting the fat composition in aquaculture fish are also of primary importance in nutritional and physiological research, where body content is traditionally determined by animal dissection followed by chemical analysis^7,8^.

In recent years, a growing number of image analysis techniques have permitted the bodies of animals to be studied in situ and so reduce the number of animals slaughtered, without a loss of precision or estimation power^4^. These techniques can be used to acquire three-dimensional information of the body with high-spatial resolution. Among them, ultrasound, magnetic resonance imaging (MRI), dual-energy X-ray absorptiometry (DEXA), photoacoustic imaging and X-ray computed tomography (CT) play an important role in the study of humans and some animals^9–12^. This work focuses on CT, a non-destructive and versatile imaging tool, which seems to be the best method among available 3D imaging techniques in terms of penetration power, attainable spatial resolution and scanning time^13^. Smaller versions of CT (Micro-CT) are currently being manufactured for the study of small animals^12–14^. Its mechanism is based on a gantry that rotates around the animal and which is located in the centre of the scanner, providing sectional images of specimens of interest without disturbing internal structures^12,14^. During CT scanning, electromagnetic radiation (X-rays) penetrates the subject from 360°. A computer software reconstructs the radiation absorbed by the tissues and its values are expressed in Hounsfield units (HU). Although this technique has been widely used to study humans and animal anatomy and assist in clinical diagnosis^12–17^, to the best of our knowledge, CT has rarely been used in aquaculture fish^4,13,18–21^. Thus, this study aims to establish the radiological values of fat density in gilthead seabream (*Sparus aurata*) in order to determine, in situ, where the fat is located and to quantify the same. Gilthead seabream is a marine species cultivated throughout the world due to its rapid growth and high survival rate, essential characteristics for the success of fish farming. To validate the method the fat content was assessed by studying changes in fed and starved fish. This paper proposes the use of micro-CT anatomical images for the evaluation of fat deposits in farmed fish.

## Results

### Fat analysis in gilthead seabream

**Figure 1:**
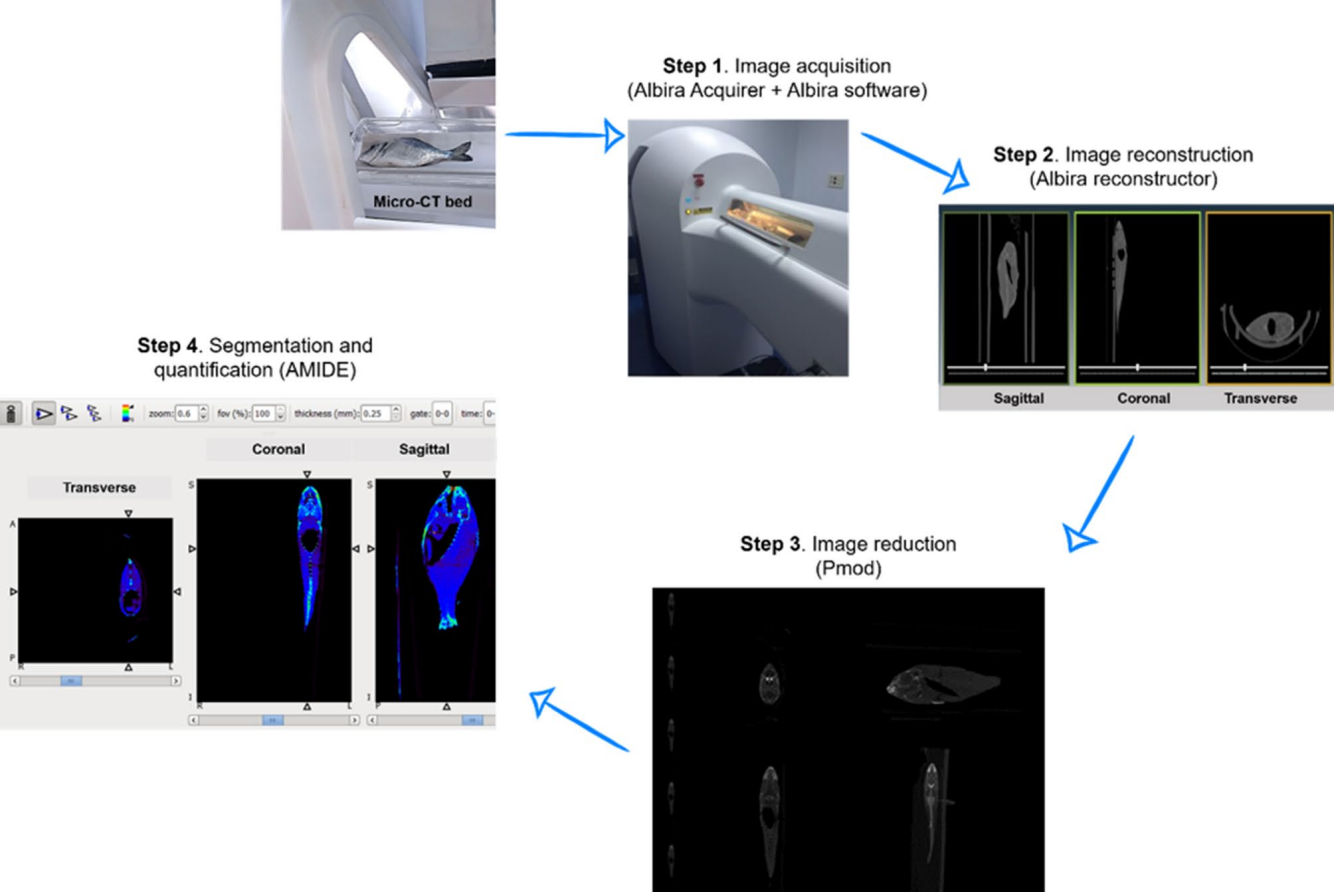
Diagram of the steps followed for the acquisition and processing of the micro-CT image in gilthead seabream specimens. (**A**) Fish is positioned in the micro-CT scanning bed and the image is acquire in micro-CT + Albira Acquirer. (**B**) Image is reconstructed in Albira reconstructor in the three spatial axes (coronal, sagittal and transverse). (**C**) Image is reduced in Pmod program. (**D**) Image analysis and quantification performing in AMIDE software.

**Figure 2:**
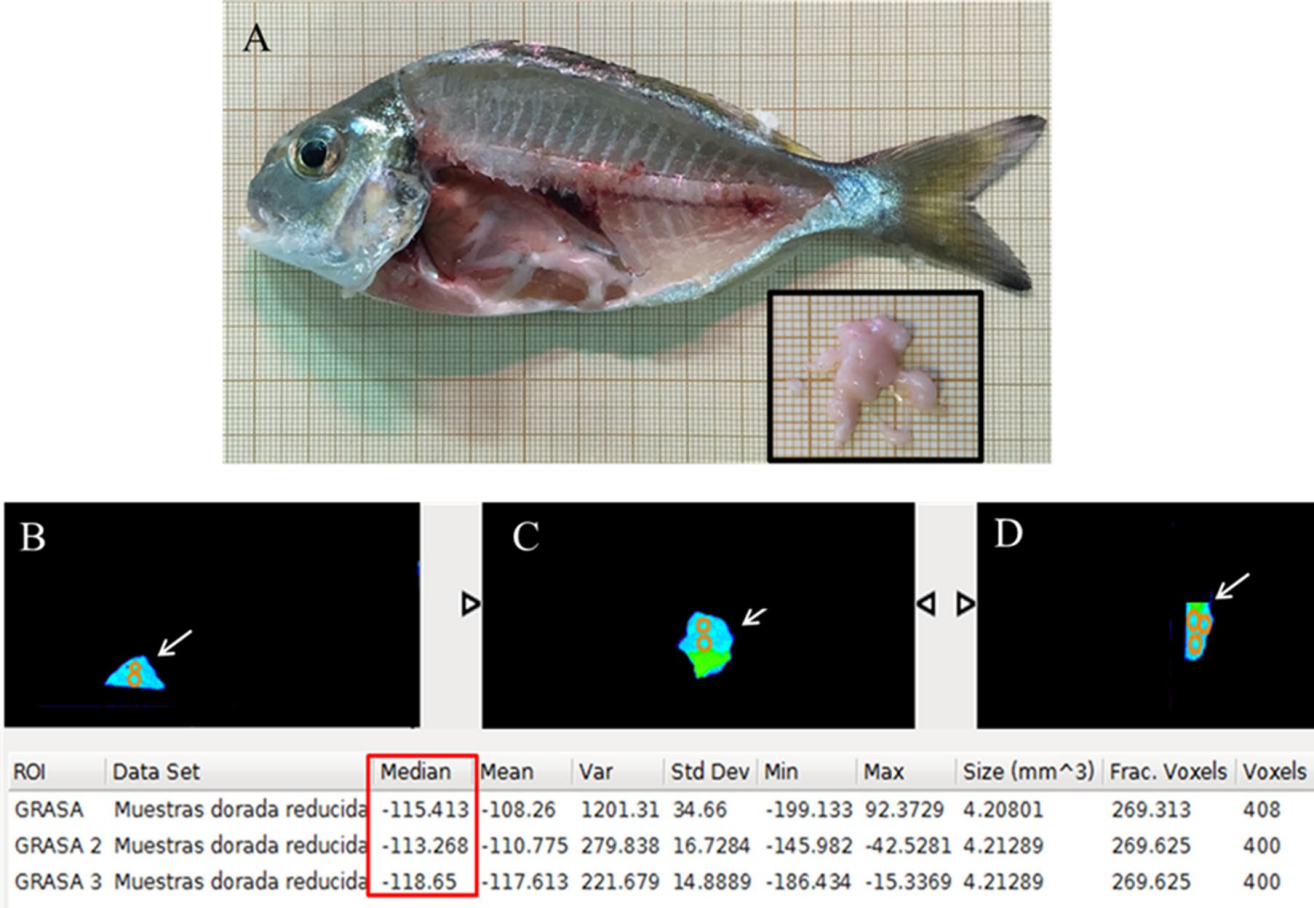
(**A**) Macroscopic image of a dissected gilthead seabream specimen with part of the muscle removed to show visceral fat localization. Inset: Visceral fat substracted from the specimen dissected in figure. Micro-CT image of visceral fat from gilthead seabream (indicated by the arrows) colored according to AMIDE analysis software and positioned on the different axes: transverse (**B**), coronal (**C**) and sagittal (**D**); 0.25 mm^3^ ROIs (orange circles) are drawn on these cuts. In the red box: mean density values for fat in the analysed voxels expressed in Hounsfield units (HU).

The main steps of acquisition and processing of the micro-CT images are illustrated in Fig. 1. The averaged density value for pure fat analyzed ex vivo was stablished in − 115 HU (Table inset in Fig. 2).

**Figure 3:**
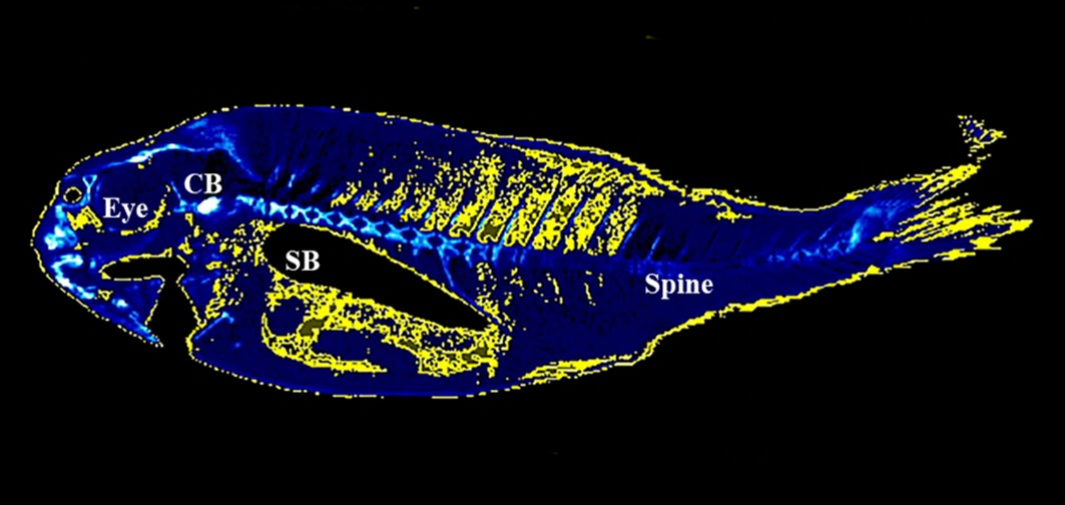
Micro-CT representative images of gilthead seabream colored in AMIDE analysis software to display the segmentation (yellow) according to the fat density range (− 115 to + 50 HU). Image is represented in sagittal axis. Eye, cleithrum bone (CB) and swim bladder (SB) are indicated as reference structures.

After studying, the micro-CT image of fish body with the AMIDE analysis software, a density range of − 115 to + 50 HU was established for fat in situ, based on the previous determination of the density value for the isolated fat from gilthead seabream. This range allowed the automated segmentation of the images by entering the values into the AMIDE software and colouring yellow the areas that coincided with the established density range (Figs. 2, 3).

### Descriptive analysis

**Figure 4:**
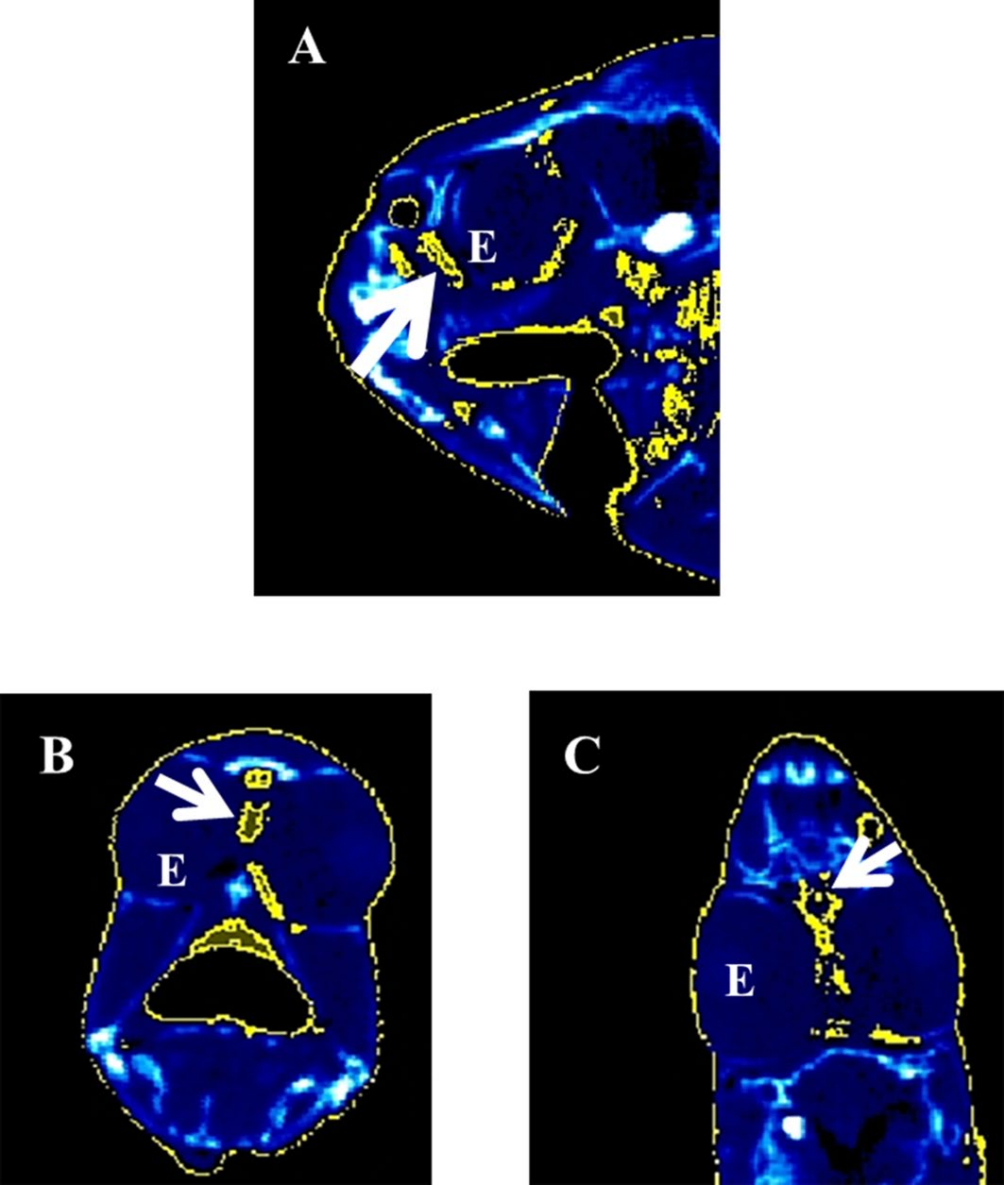
Micro-CT representative images in gilthead seabream head colored in AMIDE analysis software to display the segmentation (yellow) according to the fat density range (− 115 to + 50 HU). Image are represented in the three axes (**A**: sagittal; **B**: transverse; **C**: coronal). The eye (E) is indicated as reference structure. Arrows are indicating same areas in the three axes.

**Figure 5:**
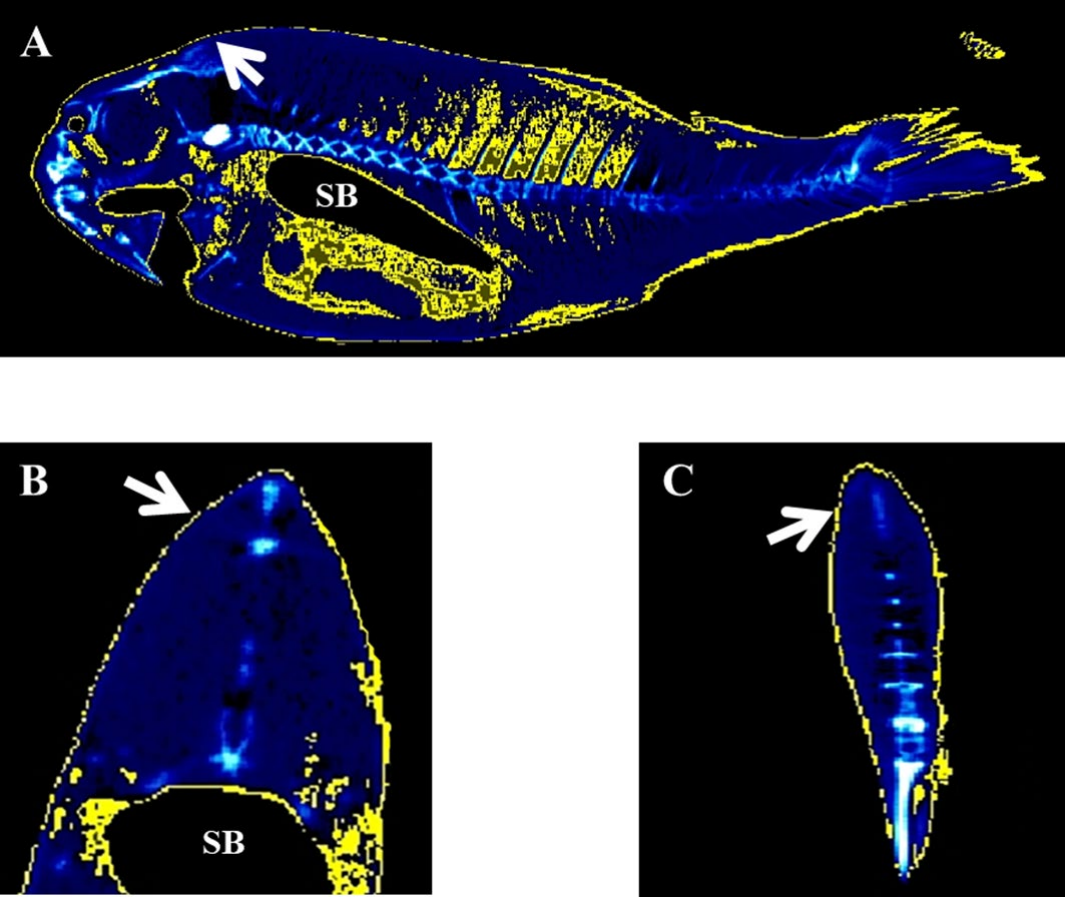
Micro-CT representative images in gilthead seabream skin colored in AMIDE analysis software to display the segmentation (yellow) according to the fat density range (− 115 to + 50 HU). Image are represented in the three axes (**A**: sagittal; **B**: transverse; **C**: coronal). The swim bladder (SB) is indicated as reference structure. Arrows are indicating same areas in the three axes.

**Figure 6:**
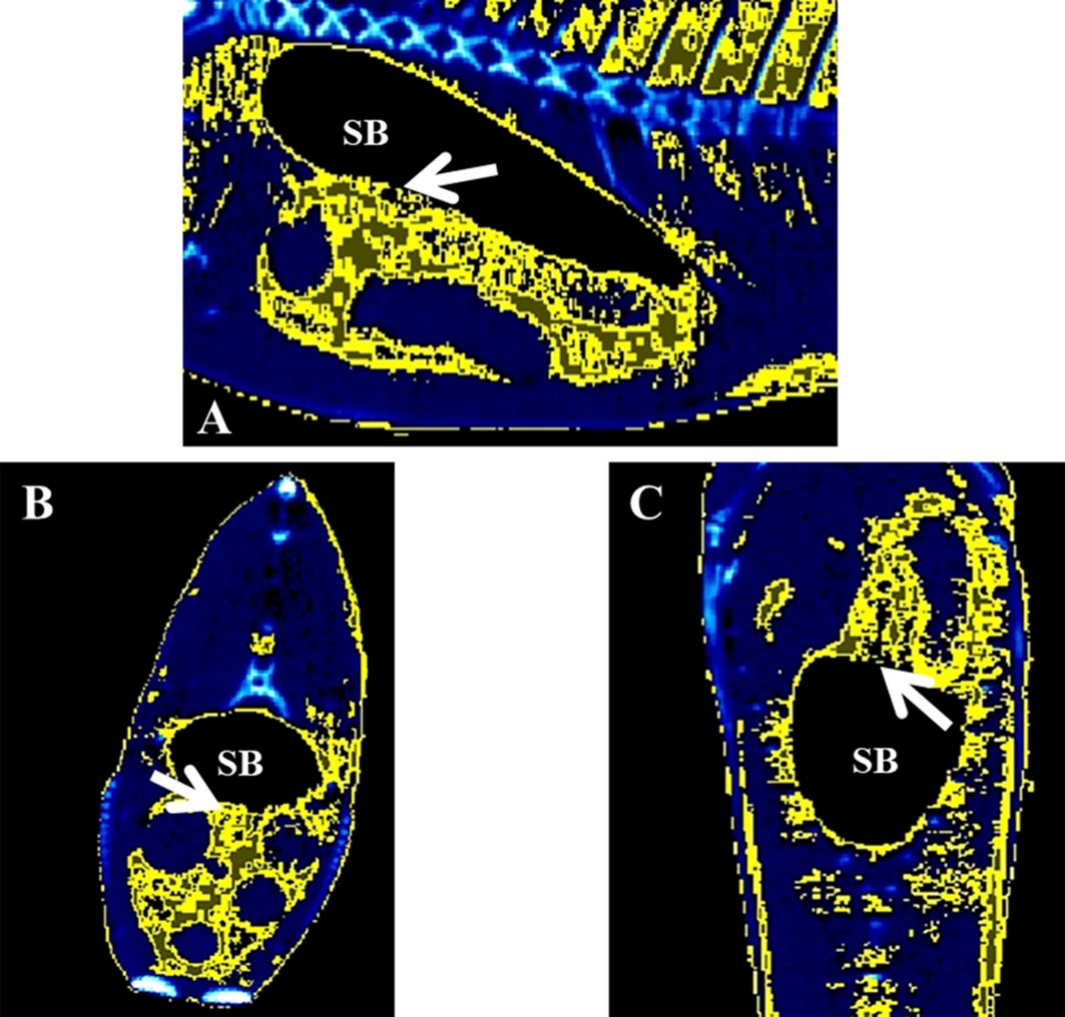
Micro-CT representative images in gilthead seabream abdominal cavity (AC) colored in AMIDE analysis software to display the segmentation (yellow) according to the fat density range (− 115 to + 50 HU).. Image are represented in the three axes (**A**: sagittal; **B**: transverse; **C**: coronal). The swim bladder (SB) is indicated as reference structure. Arrows are indicating same areas in the three axes.

**Figure 7:**
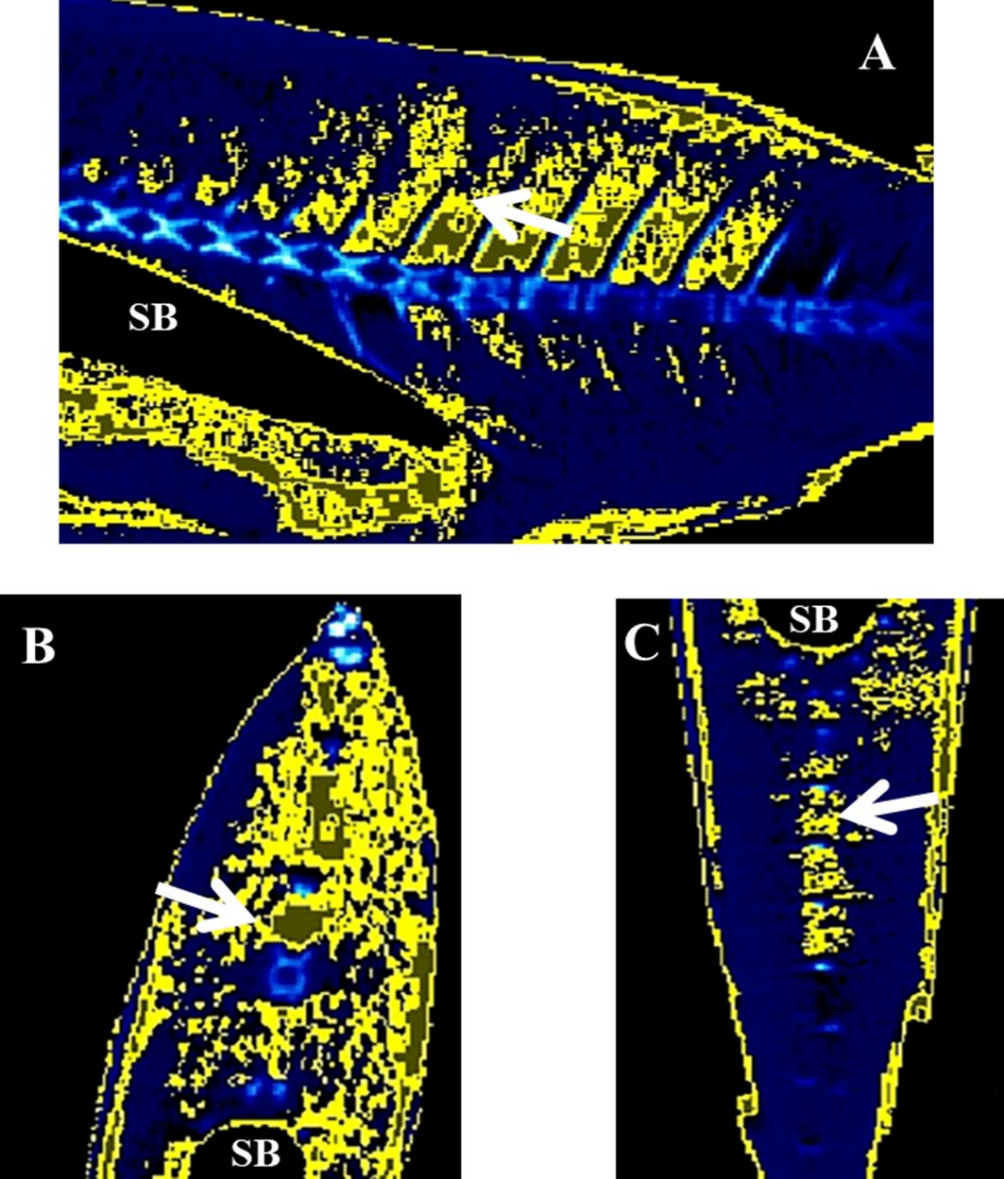
Micro-CT representative images in gilthead seabream flanks colored in AMIDE analysis software to display the segmentation (yellow) according to the fat density range (− 115 to + 50 HU). Image are represented in the three axes (**A**: sagittal; **B**: transverse; **C**: coronal). The swim bladder (SB) and spine are indicated as reference structures. Arrows are indicating same areas in the three axes.

Representative micro-CT images of the gilthead seabream in the transverse, coronal and sagittal axes are displayed in Figs. 4, 5, 6, 7. The images were segmented (yellow) according to the density range of − 115 to + 50 HU and the anatomical identification of areas where fat might exist or be deposited was studied. Segmented areas are shown in the head (including nostril, mouth, eye and operculum, Figs. 3, 4), delimiting the contour of the fish (which coincides topographically with the subcutaneous region, Fig. 5), in the abdominal cavity (Fig. 6) and in flanks (above and below the spine, Figs. 5, 6). The least segmented region can be seen in the head (Fig. 4), while in the region coinciding topographically with the abdomen and flanks segmentation is more widespread (Figs. 6 and 7).

### Quantitative analysis

AMIDE software allowed a volumetric analysis of the segmented fraction of the micro-CT images. First, the density range for the whole gilthead seabream body (− 1,000 to + 2,500 HU) was established in order to compare the fat volume with respect to the fish total volume. Our results showed that the fat volume in gilthead seabream specimens with an average weight of 26 ± 3 g and length of 12 ± 2 cm was 16.44 cm^3^, which is 48% of the fish total volume (33.72 cm^3^, Table 1).

**Table 1:** Biometric parameters of gilthead seabream specimens (*S. aurata*).

**Biometric parameters**	
Body weight (g)	26.385 ± 1.950
Body size (cm)	12.566 ± 0.625
Total volume (cm^3^)	33.792 ± 1.164
Fat volume (cm^3^)	16.442 ± 1.358
Fat related	48.737 ± 4.917
Total volume (%)	

Total volume and fat were obtained after the micro-CT acquisition and image analysis in AMIDE software. Data are represented as mean ± SD (n = 6).

### Validation of the method

**Figure 8:**
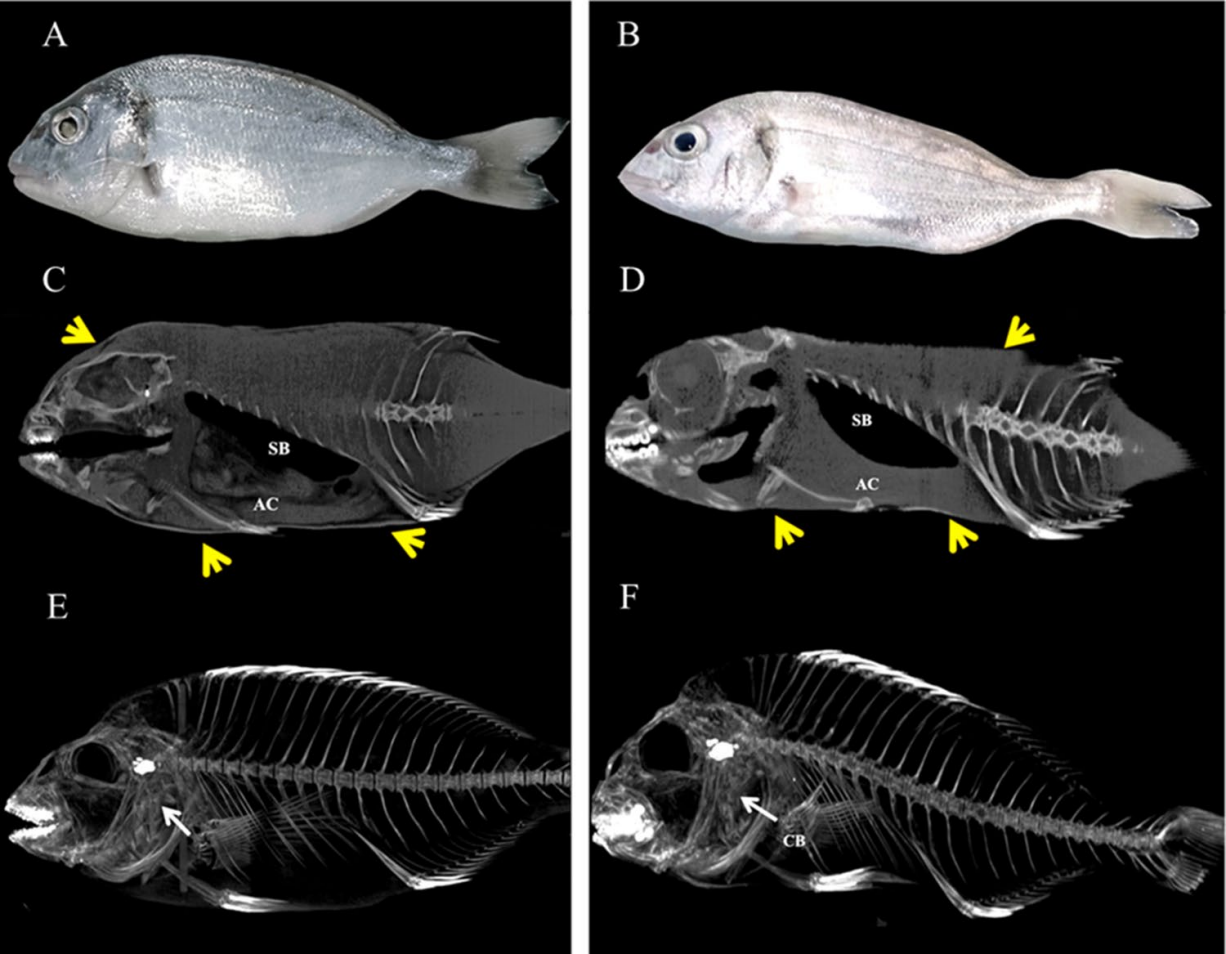
Representative images of gilthead seabream after 60 days of being fed (**A**,**C**,**E**) or under starvation (**B**,**D**,**F**) conditions. Macroscopic images of fed (**A**) and starved (**B**) fish. Micro-CT images of fed (**C**) and starved (**D**) fish, images of skeletal structure in fed (**E**) and starved (**F**) fish created in Pmod program. Yellow arrows indicate fish border, swim bladder (SB) and abdominal cavity (AC) as reference structures; white arrows indicate the cleithrum bone (CB) as the densest structure in the fish body.

To validate the method two groups of gilthead seabream specimens were kept under different nutritional conditions [fed at a rate of 1.5% body weight (control) or under starvation] for 60 days. At the end of the trial, visible differences in body dimensions were obtained (Fig. 8A,B). The differences were also evident in the micro-CT study where, as expected, greater body mass was observed in fed fish when compared to those that had been starved (Fig. 8C,D). Also, when the skeletal structure under Pmod program was acquired, visible differences were detected, mainly in the abdominal region, which appeared compressed (Fig. 8E,F).

**Figure 9:**
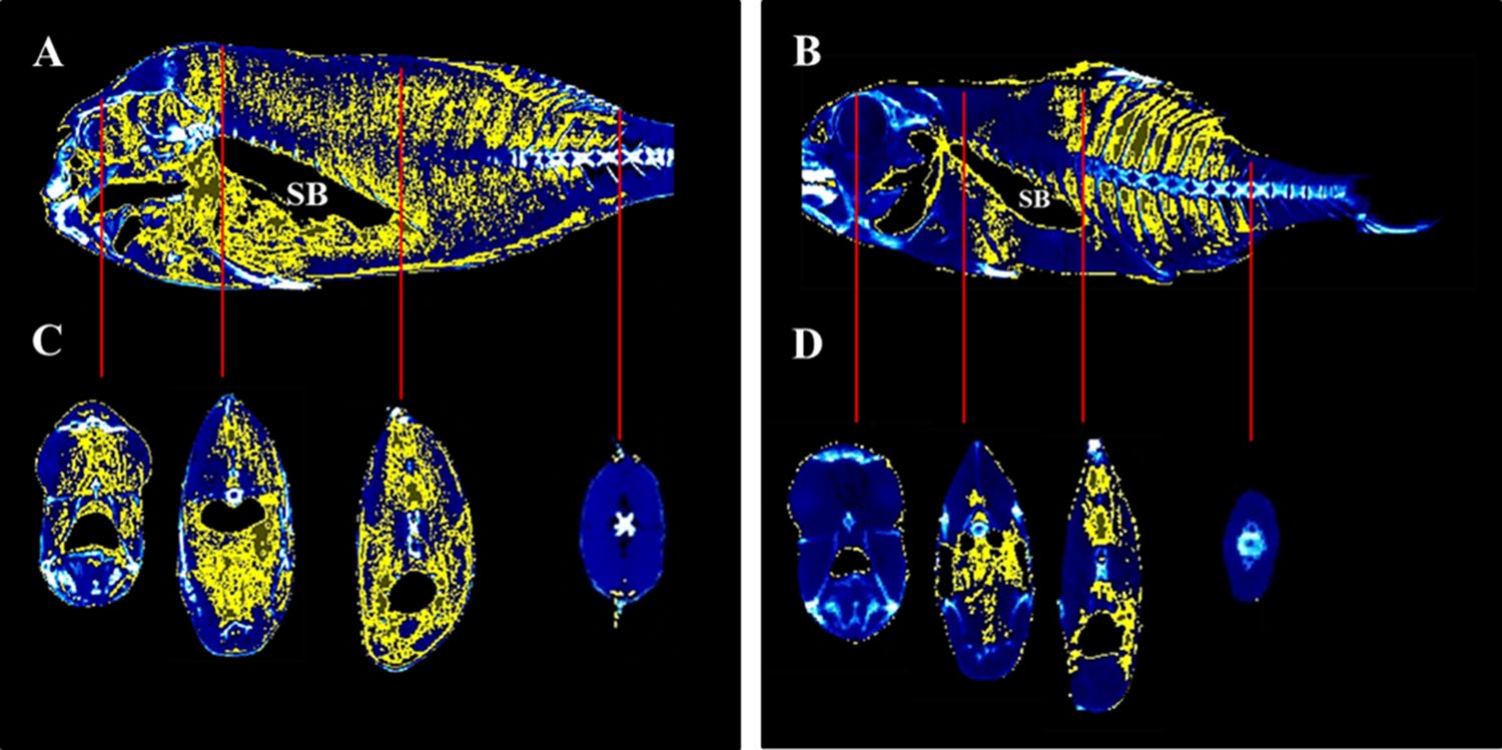
Micro-CT representative images created with AMIDE software to display fat segmentation (yellow) in gilthead seabream fed (**A**,**C**) and starved (**B**,**D**) after 60 days in sagittal and transverse axes. Red lines are connecting the location of sagittal and transverse axes. Swim bladder (SB) is indicated as reference structure.

Image analysis from fed and starved fish showed macroscopic differences between groups, more segmented areas (yellow) being found in the head (mainly in the orbital region), flanks (intramuscular) and abdominal cavity of fed fish compared to starved fish (Fig. 9). Body dimensions in starved fish were smaller than in fed fish, and the eyeball was more prominent. When biometric parameters were compared between the two groups (fed and starved), all of them showed significant differences among groups (summarized in Table 2). First, the body weight (g), length (cm) and total volume (cm^3^) of starved fish were half the values of those of fed fish (control). Curiously, the fat volume in relation to total volume was higher in starved fish (49%) than in the fed group (44%).

**Table 2:** Biometric parameters of gilthead seabream (*S. aurata*) groups fed and starved (60 days).

**Parameters**	**Experimental groups**	
	**Fed**	**Fasted**
Body weight (g)	53 ± 6.931	19.66 ± 2.200*
Body size (cm)	16 ± 1.00	12.416 ± 1.625*
Total volume (cm^3^)	71.289 ± 0.655	37.101 ± 2.077*
Fat volume (cm^3^)	31.804 ± 0.656	18.207 ± 0.774*
Fat related total volume (%)	44.618 ± 1.331	49.172 ± 3.080*

Data are represented as mean ± SD (n = 6). Statistical differences between groups are represented by an asterisk when *P* < *0.05.*

## Discussion

Aquaculture is predicted to increase more and more in forthcoming years^22^. In the case of European aquaculture, gilthead seabream (*S. aurata*) is one of the most farmed fish species^23,24^. However, to have attained this position breeding, handling and subsequent fish processing have been carefully studied. Body composition is an important trait in fish production, both from the point of view of feeding costs and consumer acceptance^4^, and, in this respect, fat plays an important role contributing to maintain or increase the nutritional and organoleptic characteristics of fish^2,5,6^. In the present work, micro-CT was used to assess fat determination in gilthead seabream, due to the above-mentioned importance of this fish in marine aquaculture.

CT assistance in assessing body composition and determining fat content in humans has been reported since several decades^25,26^. Micro-CT studies in rats and mice have been widely used to measure adipose tissue, allowing accurate volumetric quantification with high spatial discrimination^27,28^. In general, different ranges of tissue density in Hounsfield units (HU) have been established for the quantification of fat in CT studies, based on the assumption that the limits vary with the type of scanner used and that they differ between individuals or species. The most commonly used intervals are − 190 to − 30 HU and − 150 to − 50 HU. Fortunately, the variation in upper and lower limits does not have a significant effect on the assessment of fat areas^29,30^. In this study, we acquired high resolution images (125 μm) of the entire gilthead seabream and determined for the first time the HU density range for the fat (− 115 to + 50 HU, on the basis of the previously extracted pure fat) and the fat contained within the gilthead seabream body in order to enable automatic segmentation by computer software, which would allow a quantitative and descriptive analysis in situ. Previous studies in body composition by CT in a variety of fish species, *Cyprinus carpio L.*, *Ctenopharyngodon idella Val*., *Hypophthalmichtis molitrix Val*. and *Stizostedion lucioperca L.* ^4^ and *Hippoglossus hippoglossus L*. ^29^, determined different HU ranges for fat which confirm that density values can vary between species. Thus, the importance of this study lies in the possibility of characterizing radiologically the fat in gilthead seabream to provide fast and reliable results related to its content (spatial distribution, volumetric parameters or differences in density) in aquaculture systems. Another advantage of this kind of studies is that data are obtained in a comparatively short period, since the high-resolution micro-CT provides a set of precise quantitative volumetric data that would be difficult to obtain by other traditional analytical techniques^13^.

Fat distribution in different anatomical locations and in several vertebrate species, including humans, has been reported^25–28^. However, in fish the spatial distribution and measurement of fat depots have not been studied in depth. Since the presence of adipose tissue in different fish compartments also plays an important role in product quality, measurement of the fat content is a valuable information^31^. As the resolution of in vivo micro-CT can be selected to be in an isometric voxel range of approximately 10 to 200 microns, it is possible to measure not only the total volume of adipose tissue within an animal, but also to identify and quantify very small volumes of fat cells residing in discrete deposits^28^. This automatic method for fat segmentation that it has been developed for gilthead seabream is well established in murine models using computer programs similar to those used in this study^27^.

In relation to fish consumption, fat depots that interfere with carcass quality can be roughly divided into those discarded and those consumed. Discarded fat depots include the visceral fat, located in the abdominal cavity around the digestive tract (representing 2–25% of the body weight depending on fish species and the status of the fish) and the subcutaneous fat, which is located all around the body of the fish. In salmonid species, it is more prominent in dorsal or ventral zones. Dorsal subcutaneous fat is highly developed in a region situated between the head and the dorsal fin. Moreover, ventral fat is one of the components of the belly flaps localized in the abdomen of fish and represents the part of the flesh that hangs under the spines^31^. Our results showed that gilthead seabream have a similar pattern to salmonids in terms of fat location, where the presence of subcutaneous fat is higher in dorsal region and the largest area of fat segmentation is located in the abdominal cavity. In humans, the largest sites of adipose tissue deposition are either subcutaneous or intra-abdominal (within the abdominal cavity)^32,33^. On the other hand, consumed fat depots are located in white and red muscle species, with red muscle being richer in lipids than white muscle^31^.

In order to compare the fat volume related to the total volume, the density range of the whole gilthead seabream body (− 1,000 to + 2,500 HU) was also determined. Our results showed that the segmented area in the range established for the fat density in gilthead seabream represents 48% related to the total volume, which coincides with previous studies where the traditional analysis of body composition reported a high profile of fat content in this fish species^34,35^. Thus, our data raise the possibility of micro-CT being used to measure fat volume in fish in a non-destructive and reliable way.

Although there are several good reasons to accept that micro-CT scans precisely quantify fat volume based on voxel densities, the method described above was validated by comparing the segmentation results within in the range established for the fat in gilthead seabream under two different conditions (fish fed at a rate of 1.5% body weight or starved). For the present study the starved fish were food-deprived for 60 days, which can be considered a very long starvation period. The reason is that during starvation, the whole body or some organ sizes and weight can change or be significantly altered^36^. Furthermore, our interest was to provoke the mobilization of lipids, which occurs later than glycogen mobilization and is influenced by extrinsic factors such as starvation^31^.

Micro-CT images showed clear differences in body dimensions between fed and starved fish. The disposable soma theory of ageing suggests that starvation evolved as a somatic protection response to enable animals to survive periods of food shortage, by losing weight^37^. As regards meat quality, long-term starvation results in the reduction of muscle and connective tissue, protein gives an insubstantial texture to the cooked flesh^35,38^. Image analysis of the fat content of fed and starved fish pointed to a higher fat density in the cranial, subcutaneous, muscular and abdominal region of fed fish, while, in starved fish a lower distribution of fat density area was observed in those regions. The accuracy of predicting fat distribution in our study agrees with the results of previous studies that reported the influence of starvation in the mobilization of fat tissues in different compartments of fish^38,39^.

Our results showed that biometric parameters such as body weight and size differ after 60 days of feeding or starvation. Since food deprivation is a common practice in fish farming in order to regulate fish stock before marketing or before slaughtering to improve preservation^40^, several studies with long periods of food deprivation have been published to evaluate the influence of this condition on different physiological aspects^41,42^. Our results pointed to a significant reduction in body volume in starved fish compared to fed fish. It is known that quantitative and qualitative modifications of nutrients modulate the global growth of different fish species as well as overall adiposity^31^. According to our results, fat represents around 44 and 49% of total volume in fed and starved gilthead seabream, respectively. Previous studies in farmed gilthead seabream in starvation conditions reported an increase in the storage of visceral fat, indicating that a significant proportion of the fed lipids was used to produce fat tissue and lipid reserves rather than being metabolised to support growth and energy demand^43–45^, which may explain the high percentage of fat related to total body in starved fish. With innovative techniques, such as micro-CT such differences could be solved by evaluating in situ and in vivo the improvements obtained from fish feeding and management. Fortunately, non-invasive imaging techniques such as ultrasonography or infrared spectroscopy have proven to be effective in the study of fish and seafood quality^2,46^. However, these techniques have the limitation of not allowing a volumetric and three-dimensional analysis of the overall distribution and volume occupied by the fat tissue^13,46^. On the other hand, available imaging techniques such as planar dual-energy (DEXA) uses the X-rays to study body composition in a non-invasive way, furthermore demonstrating be effective to study meat composition in agriculture industry^10^. Therefore, it would be interesting to contrast this method with one of them in order to compare results and determine the most effective and economical technique to implement it in aquaculture systems or otherwise to use a combination of non-destructive techniques to strengthen the analysis. It is very important to highlight that in the micro-CT study the values in the Hounsfield scale may vary depending on the fish species, so new studies are needed to analyses fat deposits in other fish species of economic importance.

## Conclusions

This study describes a robust and reliable non-destructive method that can be used to determine and quantify fat deposits, fat content and fat infiltration into organs and tissues in gilthead seabream. According to the applied methodology and the established segmentation, we can consider that segmented areas coincide with the topographic location of these deposits and the range established for the fat (− 115 to + 50 HU). This work establishes the basis for a deeper study of fat tissue in gilthead seabream specimens, which would permit extrapolation to other aquaculture species. The proposed methodology is very precise and could contribute to the reduction of the number of sacrificed fish. Furthermore, no dissection or additional tissue processing is necessary, saving time and reducing costs.

## Materials and methods

### Animals

Eighteen male juvenile specimens (5 months) of the hermaphroditic protandrous teleost gilthead seabream (*S. aurata*) (26 ± 3 g and 12 ± 2 cm) were obtained from a local fish farm and kept in re-circulating seawater aquaria (250 L), with a flow rate of 900 L h^−1^ in the Marine Fish Facility at the University of Murcia and allowed to acclimatize for 4 weeks. A commercial diet (SKRETTING, Spain) was administered during acclimatization. The temperature and salinity were 22 ± 2 °C and 28‰, respectively. The photoperiod was of 12 h light: 12 h dark.

### Experimental trial

Fish specimens were randomly assigned into three groups (*n* = 6 each), and one group was used to determine fat distribution and biometric parameters under normal physiological conditions. Then, the rest of the fish were studied in two different conditions (fed and starved) to validate the method and ensure that the image segmentation corresponded to the fat content. The control group (fed fish) was fed with commercial pellets (SKRETTING, Spain) at a rate of 1.5% body weight day^-1^ and the last group was starved, while both groups were kept in these conditions for 60 days. All fish were euthanized by an overdose of the anaesthetic tricaine methanesulfonate (MS-222, 250 mg L^−1^, SIGMA-ALDRICH, Spain) before acquisition in the micro-CT, to establish a sensitive protocol with high image resolution and without interference from water or anaesthesia chamber. All the experimental protocols were approved by the Ethics Committee of the University of Murcia, following the guidelines of European Union for the animal handling (2010/63/EU).

### Micro-CT imaging, reconstruction and segmentation

The acquisition and processing steps of micro-CT imaging are represented in Fig. 1.

#### Imaging

All specimens were imaged at the Preclinical Imaging Facilities at the University of Murcia, using the Albira SPECT/PET/CT tri-modal preclinical-scanner (BRUKER, Karlsruhe Germany). Fish specimens were always scanned from the mouth to the caudal fin [lying on its right side on tissue paper to separate the fish from the scanning bed (115 mm length, Fig. 1)]. The X-ray source was set to a current of 200 microamps (μA) and a voltage of 45 peak kilovolts (kVp), using a 0.5 mm filter to harden the beam. A digital flat panel X-ray detector (BRUKER, Karlsruhe, Germany), with 2,400 × 2,400 pixels and a field of view of 70 × 70 mm^2^ was used to capture 600 voxel projections of 0.125 mm^3^. The total scan exposure per fish was 20–25 min. The approximate radiation deep dose equivalent for micro-CT settings was 220 milisievert (mSv) and the shallow dose equivalent was 357.4 mS. An initial calibration was performed on the basis of the visceral fat extracted from the specimens after acquisition.

#### Reconstruction

After scanning with the micro-CT, images were reconstructed in the three spatial planes (transversal, coronal and sagittal) by means the filtered back projection (FBP) algorithm in the Albira Suite 5.0 Reconstructor (BRUKER, Karlsruhe, Germany). These combined acquisition and reconstruction settings produce a final image with 125 μm isotropic voxels, which is considered, sufficient for whole animal analysis ^27^.

#### Segmentation

Images were reduced to minimize computational demands using Pmod (PMOD TECHNOLOGIES LTD, Zurich, Switzerland) and following the steps described by Loening and Gambhir^27^. Correctly reconstructed and reduced images were segmented using a free software tool for multimodality medical image analysis (AMIDE, UCLA University, LA, USA). First, we determined the density value in Hounsfield units (HU) for the pure fat by dissecting visceral fat tissue from several specimens of gilthead seabream (Fig. 2). The ex vivo fat tissue was imaged in the micro-CT and several ellipsoidal regions of interest (ROIs) of 0.25 mm^3^ size were drawn over these micro-CT images (Fig. 2B–D) in AMIDE analysis software. More than 400 voxels were analysed in each ROI to obtain the mean density value for the visceral fat in HU (Table inset in Fig. 2).

Then, to segment the fat in situ*,* 3D isocontour ROIs were manually drawn along the micro-CT image of the fish body, the HU range based on the value determined for the calibration from visceral fat subtracted was introduced in the AMIDE software and an automatic segmentation was carried out, which was coloured yellow. In cases where the density of the micro-CT bed overlapped the density of some fish portions, the value of the empty micro-CT bed was acquired to be subtracted from the fish values. To evaluate the reliability of the automatic segmentation, we compared the fat segmentation in fed fish and starved fish.

#### Image analysis

*Descriptive analysis.* Segmented images were visually observed by two experts in anatomy and radiology. A descriptive analysis in the AMIDE software was made of fish images from each experimental group. To describe the fat distribution in the fish body, we analysed the images on/along three axes (transverse, coronal and sagittal).

*Quantitative analysis*. The quantitative analysis was determined by AMIDE software through a morphometric or volumetric analysis by selecting the option “calculate ROI statistics” after automatic segmentation; this allowed us to determine parameters such as total volume and fat volume in mm^3^ (Table 1). All morphometric parameters were normalized to the total volume of the specimen, and thus are independent of the absolute size and its variation between the specimens.

### Statistical analysis

Raw data contained in each ROI isocontour (not less than 33,564) were analysed and averaged in the AMIDE analysis software for each fish. Then, the statistical analysis between groups (*n* = 6) was performed using the Statistical Package for Social Science (SPSS for WINDOWS v.23) by Student-T test. Data are presented as means ± SD (*n* = 6) and differences were considered statistically significant when P < 0.05.
